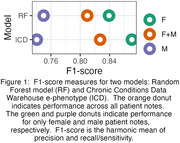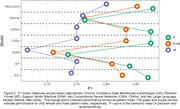# Leveraging Unstructured Clinical Notes to Improve e‐Phenotyping of Alzheimer's Disease: Insights on Gender Differences

**DOI:** 10.1002/alz70860_107112

**Published:** 2025-12-23

**Authors:** Paul Heider, Sara Knox, Stephanie Aghamoosa, Dmitry Scherbakov, Maxwell Cutty, Jihad Obeid

**Affiliations:** ^1^ Medical University of South Carolina, Charleston, SC, USA

## Abstract

**Background:**

Early identification of patients with Alzheimer's Disease and Related Dementias (ADRD) is critical for timely diagnoses and treatment. Manual chart review is labor‐intensive and impractical for large‐scale studies. The Chronic Conditions Data Warehouse (CCW) e‐phenotype is the dominant electronic health record phenotyping approach and relies on ICD‐10 codes. In this talk, we present results from exploring new methods leveraging evidence from unstructured clinical notes and identify statistically significant differences in overall model performance and individual model performance on female versus male patients.

**Method:**

We analyzed unstructured clinical notes from 1,576 patients at the Medical University of South Carolina with a balanced set of patients with and without ADRD and matched across demographics (e.g., gender). We then developed an array of models trained on 80% of the data using the CCW as a silver standard reference label. Of the remaining hold‐out test set, a gold standard ADRD determination was made through manual chart review for 200 patients. We used approximate randomization to generate 95% confidence intervals for comparing between models and for comparing between genders within models. Code for reproducing this approach is available via Codeberg: https://codeberg.org/HeiderLab/article‐addenda.

**Result:**

To highlight the type of interactions present in our study, we compare F1‐scores for the CCW and Random Forest (RF) models in Figure 1. (F1‐score reflects the harmonic mean of recall/sensitivity and precision.). The overall score for the ICD approach (F1 = 0.83) is numerically but not significantly higher than for the RF approach (F1 = 0.81). The margin of difference between genders is significant for the ICD results but not for the RF results. Thus, the ICD approach is better on average but also significantly biased. As shown in Figure 2, all models had numerically higher F1‐scores on female notes despite the balanced corpus. We are investigating the underlying cause of these trends.

**Conclusion:**

We also analyzed AUC, accuracy, specificity, precision, and recall/sensitivity. No single model outperformed other models across all measures. Researchers must consider the context of the use for e‐phenotyping to determine if, e.g., high recall/sensitivity or high specificity is more important. Further considerations like disaggregated performance by demographics are also important deciders.